# Keyboarding Instruction for a Japanese Child With Attention Deficit Hyperactivity Disorder and Dysgraphia Reduced Frustration in Handwriting: A Case Report

**DOI:** 10.7759/cureus.74298

**Published:** 2024-11-23

**Authors:** Katsuya Nakamura, Shinsuke Nagami, Daichi Iimura, Masashi Shiomi

**Affiliations:** 1 Department of Speech-Language Pathology and Audiology, Faculty of Rehabilitation, Kawasaki University of Medical Welfare, Kurashiki, JPN; 2 Department of Communication Disorders, School of Rehabilitation Sciences, Health Sciences University of Hokkaido, Ishikari, JPN; 3 Institute of Human Sciences, University of Tsukuba, Tsukuba, JPN

**Keywords:** attention deficit, frustration, handwriting, hyperactivity disorder, information and communication technologies

## Abstract

Dysgraphia often goes unnoticed in schools, leading to delayed academic development and diminished self-esteem for affected students. This case report provides keyboarding instruction to a nine-year-old Japanese boy diagnosed with dysgraphia and observes its impact on his writing performance, including speed, accuracy, and composition, and mental burden. The patient was diagnosed with dysgraphia and refusal to write at school. We conducted an initial assessment to confirm dysgraphia and identify the student’s reluctance to write in a school setting. Subsequently, we administered keyboarding instruction sessions two to three times a month, each lasting 20-40 minutes. These sessions emphasized typing skills and keyboard shortcuts. We assessed the mental burden associated with handwriting and keyboarding using a visual analog scale (VAS). We also tracked his keyboarding speed across sessions to monitor his progress. VAS scores were clearly lower for keyboard input compared to handwriting. His keyboarding speed improved with each session, eventually surpassing his handwriting speed. Keyboarding enhanced accuracy, reduced errors and revisions, and showed superior kanji ability. Our findings suggest that keyboarding instruction can alleviate frustration and foster positive learning attitudes among Japanese children with dysgraphia. This report underscores the feasibility and effectiveness of implementing keyboarding as an intervention for students with dysgraphia, potentially decreasing their frustration and enhancing their participation in classroom activities. The progressive improvement in keyboarding speed highlights the importance of continued practice and support for achieving better educational outcomes.

## Introduction

Common symptoms of dysgraphia include difficulties with letter formation, spacing, and fluency in handwriting, persisting into their school-age years [1]. The core symptoms of attention deficit hyperactivity disorder (ADHD) include inattention, impulsivity, and hyperactivity. Children with ADHD often experience ongoing academic challenges, particularly with handwriting performance [1,2]. Additionally, children with fine motor difficulties, such as developmental coordination disorder (DCD), frequently grapple with difficulties in manual writing [2]. Handwriting is an essential skill to be acquired during elementary education, consuming a substantial portion of children’s school time [3].

However, children with dysgraphia, despite their education, may struggle to attain automatic handwriting skills [4]. Failing to acquire automatic handwriting can lead to challenges in handling concurrent tasks, such as grammar, spelling, and composition, which become increasingly demanding as students progress through grades. These difficulties can result in heightened fatigue, frustration, diminished cognitive capabilities, and lowered self-esteem among children with dysgraphia [5]. Consequently, these children tend to avoid writing tasks over time, which potentially leads to their delayed academic development and school refusal [5]. Detecting dysgraphia early and providing appropriate learning support is imperative.

For children grappling with dysgraphia, digital tools, particularly computers, have been employed to facilitate learning [6,7]. Previous interventions have encompassed handwriting practice, relaxation techniques, and sensory-based training, often administered by occupational therapists, to enhance handwriting legibility [8]. A systematic review of such interventions recommends a minimum of two handwriting practice sessions per week, totaling at least 20 sessions, to enhance handwriting skills [8]. Nevertheless, the effectiveness of these interventions in improving handwriting speed remains unproven, potentially necessitating more frequent and extended practice [8]. Enhanced intensity of handwriting practice appears to be a requirement for improved handwriting performance. It is important to acknowledge that the mental burden associated with dysgraphia might persist despite these interventions.

In line with the advancement of digital technologies, alternative approaches to written tasks have gained prominence [6,9]. Keyboarding instruction, compared with traditional handwriting, has the potential to enhance writing performance, such as speed, accuracy, and composition among children with dysgraphia [6]. Keyboarding may reduce the fine motor demands associated with handwriting, potentially enabling faster and more accurate expression. Despite the growing body of evidence supporting the efficacy of keyboarding instruction, few studies focus on its application in the context of the Japanese language and its unique writing systems [10]. The Japanese language uses two syllabic systems (hiragana and katakana) and an ideographic system (kanji), which differ significantly from the English alphabetic system. It remains unclear whether findings from keyboarding instruction studies for children with dysgraphia, using the English alphabetic system, can be generalized to Japanese children with dysgraphia.

This case report aims to address these gaps by conducting keyboarding instruction lessons for a nine-year-old Japanese boy diagnosed with both ADHD and dysgraphia. Building upon existing research on keyboarding instruction, we hypothesize that keyboarding instruction may lead to improved writing performance in Japanese children with dysgraphia compared to traditional handwriting. We also hypothesize that a keyboard can serve as an assistive technology capable of alleviating these children’s mental burdens associated with writing tasks.

## Case presentation

The patient, a nine-year-old right-handed boy in fourth grade, was assessed for dysgraphia and refusal to write at school. A speech-language-hearing therapist (SLHT) provided support for his writing difficulties at our hospital. Both parents had no issues with reading or writing, and family relations were excellent. At age six, his teacher observed emotional control issues, impulsiveness, verbal aggression, difficulty in group settings, and an inability to sit still. He was diagnosed with ADHD (Diagnostic and Statistical Manual of Mental Disorders, 5th Edition) and treated with methylphenidate, which was discontinued due to lack of effect. By age seven, he attended support classrooms for some subjects, and by age nine, he was in support classrooms for all subjects. His posture during training was poor and sustained postural retention was difficult. ADHD rating scale [11] revealed a score of 26 points (10 inattentive and 16 hyperactive-impulsive). The Developmental Coordination Disorder Checklist [12] revealed a score of 42 points (fine motor: 23/40, gross motor: 13/25, and catch and throw: 6/10).

**Figure 1:**
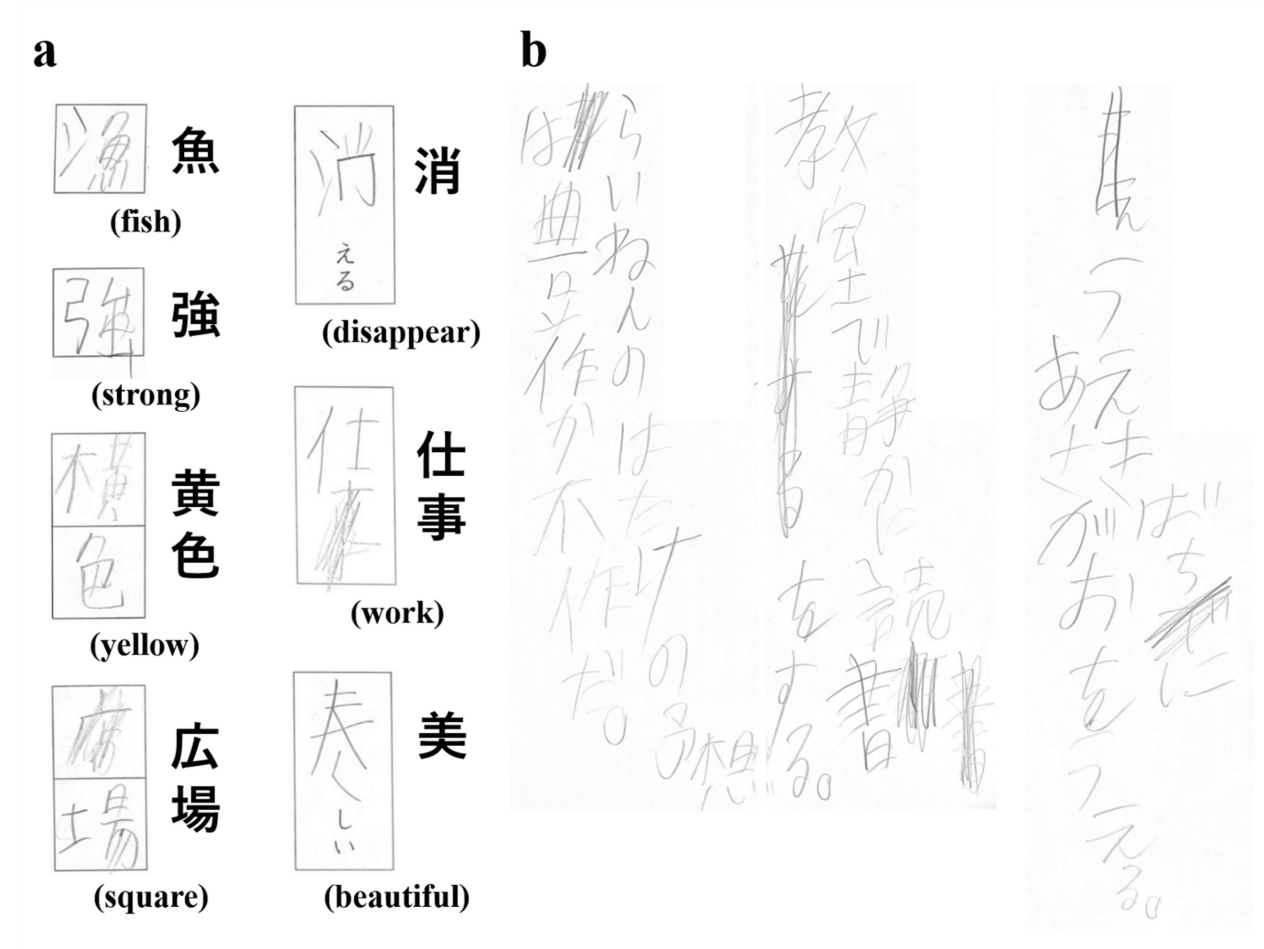
Sample of handwriting. (a) The handwriting sample on the left shows the student’s attempt to write the words. Correct words are displayed on the right, and their meanings are indicated in parentheses. (b) Despite no grammatical errors in the student’s writing, the arrangement of sentences is distorted and often rewritten.

Table 1 presents the results of the psychological assessments, which collectively indicate that the boy was diagnosed with dysgraphia, without any indications of reading disabilities. Figure 1 presents a sample of the handwriting.

**Table 1:** Student’s characteristics. WISC-Ⅳ: Wechsler Intelligence Scale for Children – Fourth edition; FSIQ: Full-Scale Intelligence Quotient; VCI: Verbal Comprehension Index; PRI: Perceptual Reasoning Index; WMI: Working Memory Index; PSI: Processing Speed Index; K-ABCⅡ-J: Kaufman Assessment Battery for Children, Second Edition, Japanese version; MPI: Mental Process Index; AcI: Achievement Index; URAWSS-Ⅱ: Understanding Reading and Writing Skills of School Children Ⅱ; ROCFT: Rey-Osterrieth Complex Figure Test.

Psychological assessments	Results
WISC-Ⅳ	
FSIQ	108
VCI	119
PRI	100
WMI	106
PSI	96
K-ABCⅡ-J	
MPI	118
Sequential	112
Simultaneous	107
Planning	116
Learning	119
AcI	128
Vocabulary	146
Reading	124
Writing	87
Mathematics	125
URAWSS-Ⅱ	
Writing speed (characters/min)	20.3
Reading speed (characters/min)	543
ROCFT	
Copy trial (scores)	22.5
Recall trial (scores)	Not measurable
Delay trial (scores)	Not measurable

Therapeutic intervention

With the consent of the participant’s mother, we initiated keyboarding training, positioning it as an alternative to traditional handwriting. A laptop was employed for this purpose, configured for roman input. During sessions, the participant was seated in a chair equipped with a backrest, with the keyboard and monitor positioned directly in front of his body. Although existing studies suggest the provision of 25-30 hours of keyboarding instruction, consistent evidence regarding specific keyboarding programs is limited [7]. Therefore, we conducted keyboarding instruction sessions two to three times a month, each spanning 20-40 minutes, with breaks integrated as necessary to accommodate his concentration and fatigue levels. The keyboarding training encompassed both typing training (TT) and keyboard shortcuts training (KST).

Typing Training

TT involved the use of web-based texts at the participant’s grade level, accessible at https://happylilac.net/syogaku.html [13]. He was tasked with typing the same text in Japanese, comprising hiragana, katakana, and kanji characters. Hiragana characters could represent either a single vowel or a combination of consonants and vowels. When entering hiragana words in Japanese, the participant needed to recall the corresponding letters for each hiragana character. For katakana or kanji words, he was required to press the conversion key after inputting the hiragana words. Initially, he practiced typing a selected text for 30 seconds as a warm-up exercise. Subsequently, he was instructed to type the text as swiftly and accurately as possible for a three-minute duration. Additionally, he was encouraged to correct any errors encountered during typing.

Keyboard Shortcuts Training

KST centered on instructing the patient in the use of common shortcut keys (copy: Ctrl + C; paste: Ctrl + V; save: Ctrl + S; screenshot: PrtSc). To facilitate this, the participant was given a printout containing instructions for each keyboard shortcut, followed by practical exercises to reinforce the concepts. During these exercises, the participant practiced employing each keyboard shortcut, carefully verifying their application. Specifically, he repeatedly performed actions, such as copying and pasting text and images using these keyboard shortcuts. The save shortcut was employed to preserve documents related to both TT and KST. Furthermore, the screenshot key was utilized to capture specific screens, with subsequent pasting and cropping of the image to fit predefined frames. Following the acquisition of these keyboard shortcuts, the participant consistently employed them to conduct various tasks, including responding to questions and inserting images from internet searches.

Reasonable accommodation in school

Instead of taking notes in class, class materials were provided as images on a tablet for him to access at home. He was also permitted to use a keyboard instead of writing by hand whenever he wished.

Outcome measurements

The assessment of the mental burden associated with both handwriting and keyboarding tasks was conducted using a visual analog scale (VAS) [14]. To represent the mental burden scale, participants were instructed to make a mark on a 10 cm horizontal line located at the center of an A4-sized paper. The position of this mark on the scale quantified the intensity of the mental burden, with greater length along the line indicating a more pronounced mental burden.

Handwriting and keyboarding speeds (characters/min) were evaluated using the Understanding Reading and Writing Skills of Schoolchildren II (URAWSS-II) [15]. Several derived indicators were calculated, including the number of errors, the number of typed characters, the number of corrections, and transcription accuracy. Kanji ability was assessed by calculating the percentage of correct answers using a web-based educational learning printout (https://happylilac.net/syogaku.html (in Japanese)) [13].

TT records documented the number of inputs (defined as the number of characters correctly typed in response to the sample) and deletions (defined as the number of deleted characters). Keyboarding speed (characters per minute) was computed as the total number of inputs divided by three minutes. A Spearman’s rank correlation test was conducted to analyze the correlation between the keyboarding speed and the number of sessions. A p-value of <0.05 was considered statistically significant.

Informed consent was obtained from both the participant and his mother. The study protocol was approved by the Ethics Committee of the Kawasaki Medical School in Okayama, Japan (Approval No.: 6387-00) and conducted per the Helsinki Declaration.

Results

Table 2 shows the outcome measurements from the first training session for both handwriting and keyboarding. The VAS indicated a significantly higher mental burden for handwriting, while the burden for keyboarding was much lower. The participant preferred using the keyboard and stated after three months, "I’m good at keyboarding."

**Table 2:** Comparison of handwriting and keyboarding outcome measurements in first training session. VAS: visual analog scale; URAWSS-Ⅱ: Understanding Reading and Writing Skills of School Children Ⅱ. Note: A longer length on the scale indicates a stronger mental burden in VAS.

	Handwriting	Keyboarding
VAS (cm)	12.5	1.7
URAWSS-Ⅱ		
Transcription speed (characters/min)	20.3	13
Total characters (characters)	61	39
Mistakes in the number of characters (characters)	2	0
Number of revisions (times)	13	0
Transcription accuracy (%)	78.7	100
Kanji ability (%)	－	100

**Figure 2:**
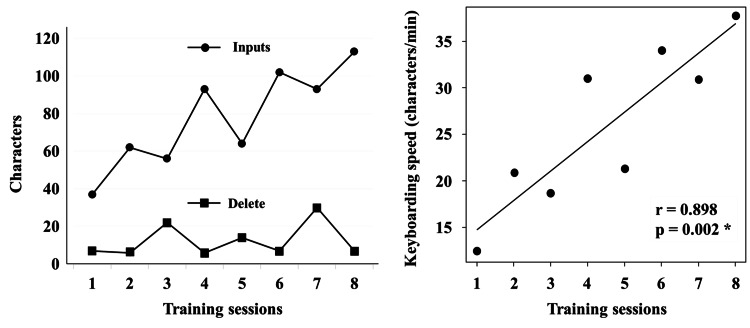
Typing training progress and correlation analysis. (a) Progress in typing training. (b) Correlation between the number of sessions and keyboarding speed. * P < 0.005.

Keyboarding was initially slower than handwriting, but with fewer errors and corrections (Table 2), TT showed a strong positive correlation between the number of sessions and speed (r = 0.898, p = 0.002) (Figure 2). By the eighth session, keyboarding speed (37.67 characters/min) exceeded the handwriting speed (20.30 characters/min). Furthermore, he showed proficiency in smoothly inputting kanji characters using the keyboard and successfully completed all questions.

He decided to attend a regular classroom in the new school term and was able to remain there, although he was occasionally absent due to a lack of motivation.

## Discussion

In this case report, we administered keyboarding training, including TT and KST, to a young boy with dysgraphia. This intervention revealed two key insights. First, keyboarding was less mental burden compared to handwriting. Second, keyboarding enhanced accuracy, reduced errors and revisions, and developed superior kanji abilities. Initially, the transcription speed for keyboarding was slower, but it improved with each session. These results suggest that keyboarding can alleviate the cognitive and emotional challenges faced by children with dysgraphia and enhance their learning attitude.

Keyboarding may be an effective assistive technology to reduce frustration with handwriting. Dysgraphia often leads to frustration and writing avoidance, negatively impacting academic performance [5]. Unfortunately, students with dysgraphia may be viewed by teachers as lazy and unmotivated, which can lead to low self-esteem and behavioral problems. To solve these problems, early assessment and intervention for dysgraphia in schools are necessary to reduce students’ frustration with handwriting.

Keyboarding can be a valuable tool for improving writing performance in students with handwriting difficulties, enhancing accuracy, productivity, and overall writing quality. Keyboarding instruction was provided to improve accuracy, speed, and touch-keyboarding skills [6]. Writing by hand and keyboarding require different sets of skills [16]. Through keyboarding, students with handwriting difficulties may improve their writing performance, including accuracy, productivity, and writing quality [6,7]. On the other hand, a previous study suggested that keyboarding decreases speed and affects the quality of composition more than handwriting [6]. As an effective alternative to handwriting in schools, it is recommended that students be able to keyboard at least as fast as they can handwrite [16]. Therefore, interventions to improve work efficiency, such as KST, are important to compensate for keyboarding speed. Notably, the student gradually improved his keyboarding speed across the training sessions, eventually surpassing his handwriting speed while requiring minimal deletions.

In educational settings, keyboarding as an alternative to handwriting for students with dysgraphia may reduce frustration. Dysgraphia often co-occurs with ADHD and DCD, characterized by handwriting difficulties [1,2]. The student's dysgraphia, without reading difficulties, may result from ADHD, DCD, and poor visuospatial constructional ability. Dysgraphia persists throughout elementary school, significantly affecting academic performance, and highlighting the need for early remediation and compensatory strategies [17]. Schools often overlook assessment and intervention for dysgraphia [9]. This study employed keyboarding instruction successfully, reducing writing-related frustration, and improving participation and writing performance, including accuracy and kanji skills. Consistent keyboarding training can reduce frustration and improve performance for students with dysgraphia. Early assessments and individualized programs are crucial. Teachers should support these students, and accommodations like keyboard use for written tasks should be implemented to facilitate learning and participation.

However, this report has limitations. It is based on a single case, making broader applicability challenging. Longitudinal studies are required to examine the impact of keyboarding on mental burden. Further research is required to investigate the effectiveness of keyboarding instruction. The long-term effects of keyboarding interventions on academic performance, learning attitudes, and handwriting were not examined. Future research should focus on longitudinal studies and explore tailoring interventions for different dysgraphia subtypes. This will help educators integrate keyboarding training effectively and provide comprehensive support for students facing handwriting challenges. This report underscores the potential of keyboarding to reduce frustration and enhance learning attitudes for Japanese children with dysgraphia.

## Conclusions

This single case report suggests that keyboarding instruction can help reduce frustration and foster positive learning attitudes in Japanese children with dysgraphia. The report highlights the feasibility and effectiveness of implementing keyboarding as an intervention for students with dysgraphia, potentially decreasing frustration and enhancing classroom participation. While these findings suggest the potential benefits of such interventions, they should not be generalized beyond this case. Further research with larger samples is necessary to confirm these outcomes and explore the broader applicability of keyboarding as an educational tool for students with handwriting difficulties.
